# Performance Investigation of PSF-nAC Composite Ultrafiltration Membrane for Protein Separation

**DOI:** 10.3390/polym16182654

**Published:** 2024-09-20

**Authors:** Gunawan Setia Prihandana, Muslim Mahardika, Budi Arifvianto, Ario Sunar Baskoro, Yudan Whulanza, Tutik Sriani, Farazila Yusof

**Affiliations:** 1 Department of Industrial Engineering, Faculty of Advanced Technology and Multidiscipline, Universitas Airlangga, Jl. Dr. Ir. H. Soekarno, Surabaya 60115, Indonesia; 2 Department of Mechanical and Industrial Engineering, Faculty of Engineering, Universitas Gadjah Mada, Jalan Grafika No. 2, Yogyakarta 55281, Indonesia; muslim_mahardika@ugm.ac.id (M.M.); budi.arif@ugm.ac.id (B.A.); 3 Department of Mechanical Engineering, Faculty of Engineering, Universitas Indonesia, Kampus Baru UI, Depok 16424, Indonesia; ario@eng.ui.ac.id (A.S.B.); yudan@eng.ui.ac.id (Y.W.); 4 Department of Research and Development, P.T Global Meditek Utama-IITOYA, Sardonoharjo, Ngaglik, Sleman, Yogyakarta 55581, Indonesia; tsriani@iitoya.com; 5 Centre of Advanced Manufacturing & Material Processing (AMMP Centre), Department of Mechanical Engineering, Faculty of Engineering, Universiti Malaya, Kuala Lumpur 50603, Malaysia; farazila@um.edu.my; 6 Centre for Foundation Studies in Science, University Malaya, Kuala Lumpur 50603, Malaysia

**Keywords:** polysulfone, clean water, membranes, nano-activated carbon, good health, protein separation

## Abstract

As a promising wastewater treatment technology, ultrafiltration membranes face challenges related to fouling and flux reduction. To enhance these membranes, various strategies have been explored. Among them, the incorporation of nano-activated carbon (nAC) powder has emerged as an effective method. In this study, composite polysulfone (PSF) ultrafiltration membranes were fabricated using nAC powder at concentrations ranging from 0 to 8 wt.%. These membranes underwent comprehensive investigation, including assessments of membrane morphology, hydrophilicity, pure water flux, equilibrium water content, porosity, average pore size, and protein separation. The addition of activated carbon improved several desirable properties. Specifically, the hydrophilicity of the PSF membranes was enhanced, with the contact angle reduced from 69° to 58° for 8 wt.% of nAC composite membranes compared to the pristine PSF membrane. Furthermore, the water flux test revealed that 6 wt.% activated carbon-based membranes exhibited the highest flux, with a nearly 3 times improvement at 2 bar. Importantly, this enhancement did not compromise the protein rejection. Additionally, the introduction of nAC had a significant effect on the membrane’s pore size by improving lysozyme rejection up to 40%. Overall, these findings will guide the selection of the optimal concentration of nAC for PSF ultrafiltration membranes.

## 1. Introduction

Membrane technology finds extensive applications across various fields, including agri-food, biomedicine, and research. Researchers have developed several methods, such as protein adsorbents and capillary electrophoresis, to effectively separate protein mixtures [1,2]. Membrane separation is widely recognized as an efficient method due to its low energy consumption, high efficiency, and environmentally friendly properties [3,4]. Ultrafiltration (UF) membranes play a crucial role in biotechnological applications. They are widely employed for protein filtration and separation [5,6], including in tasks such as enzyme extraction [7] and concentrating proteinaceous solutions [8]. In contemporary membrane technology, polymers and ceramics serve as the primary materials for membrane fabrication. However, due to their ease of forming into various shapes and inherent flexibility, polymeric membranes outperform ceramic counterparts [9,10].

Researchers have investigated multiple polymers, including polysulfone [11], polyethersulfone [12], polyetherimide [13], and polyvinylidene fluoride [14], which find widespread use in the production of filtration membranes. Among various polymers, polysulfone (PSF) is widely acknowledged as one of the most commonly used materials for fabricating polymeric membranes, owing to its exceptional mechanical properties, chemical resistance, and thermal stability [15,16]. Unfortunately, due to their surface hydrophobicity, PSF membranes are more susceptible to fouling, resulting in lower flux and thus limiting their further applications [17]. Therefore, in recent years, efforts have been directed toward developing membrane hydrophilicity, which directly affects improved flux rates and greater selectivity [18,19]. Incorporating hydrophilic materials [20], modifying both the surface [21] and internal structure [22], has been extensively implemented due to ease of fabrication and significant enhancement in separation performance. Different types of fillers, including metals [23], carbon materials [24,25], and zeolite [26], have been used as modifiers in the preparation of polymeric membranes.

Gavabari et al. [27] assessed the efficiency of a porous and flexible nanocomposite based on polyurethane, which incorporates activated carbon and cellulose nano-whiskers (CNWs), in terms of adsorbing methylene blue (MB) and basic violet16 (BV16). It was found that the PU nanocomposite exhibits an adsorption mechanism for MB and BV16 dyes that combines both chemical and physical processes. Furthermore, the water contact angle tests demonstrated that the inclusion of 1.0 wt.% CNW led to the greatest hydrophilicity in the nanocomposite film. Alsalhy et al. [28] fabricated an anti-biofouling polyvinyl chloride/zinc oxide (PVC/ZnO) membrane using the phase precipitation method for application in a submerged membrane bioreactor for hospital wastewater treatment. By incorporating ZnO nanoparticles in varying amounts (0.1, 0.2, 0.3, and 0.4 g), the structural morphology and hydrophilicity of the membrane were positively affected, leading to an enhancement in pure water flux.

The incorporation of activated carbon (AC) in UF has increasingly attracted interest in the last ten years. This technology offers advantages such as enhanced performance and cost competitiveness in comparison to alternative membrane alteration techniques. No consensus exists at present on the appropriate size of activated carbon selection criteria for improving the performance of polymeric membranes. Integrating adsorbents like activated carbon into membranes can improve their performance and substantially reduce membrane fouling [29]. Choi et al. [30] synthesized polysulfone multi-walled carbon nanotube membranes in which the incorporation of carboxylic functionalized carbon nanotubes resulted in increased surface hydrophilicity. The characteristics of nano-AC, such as its hydrophilicity and large surface area, would lead to the development of a nanocomposite that exhibits enhanced flux and selectivity in ultrafiltration membranes. While numerous works have examined the incorporation of nAC into polymeric membranes, the primary emphasis has been on the efficacy of removing certain components, such as turbidity and color, in water treatment. The influence of AC on protein separation has received minimal attention. Since an excessive amount of AC may have detrimental effects on the membrane by increasing sludge viscosity, it is vital to optimize the concentration of AC in membranes.

Hence, the objective of this work is to systematically investigate the utilization of nano-activated carbon (nAC) powder as a filler in the production of PSF mixed-matrix membranes using a novel approach involving blending low to high nAC concentrations into polymer membranes for protein separation. The prepared membranes underwent comprehensive characterization using analytical tools such as SEM, pure water flux, porosity, average pore size, protein separation, and water contact angle. The performance of the fabricated PSF mixed-matrix membranes was subsequently assessed through measurements of pure water flux and protein rejection.

## 2. Materials and Methods

### 2.1. Materials

Polysulfone with a molecular weight of 150,000 was acquired from P.T. Solvay Chemicals, Cilegon, Indonesia. We obtained 50 nm nAC from Nanostructured & Amorphous Materials, Inc., Katy, TX, USA, with specific surface area (SSA) of 500 m^2^/g, bulk density of 3.02–3.30 g/cm^3^, and aerodynamic particle sizer (APS) less than 50 nm. All nAC data were provided by the manufacturer. N-methylpyrrolidone (NMP) at a purity of 99.5% was sourced from Merck KGaA, Darmstadt, Germany. Additionally, we purchased lysozyme (12 kDa), pepsin (35 kDa), and bovine serum albumin (BSA) (69 kDa) proteins from HiMedia Ltd., Mumbai, India. All the chemical reagents were of analytical grade and used without further purification. Pure water was prepared using a water purification system.

### 2.2. Membrane Fabrication

The wet phase inversion technique was utilized to fabricate the membranes. In a standard procedure, a mixture of 20 wt.% PSF and nAC at varying concentrations (0, 2, 4, 6, and 8 wt.%) was dissolved in NMP, as shown in Table 1. The solution was vigorously agitated and subjected to constant heat for 12 h at a temperature of 80 °C in order to attain uniformity. Subsequently, the membrane solutions were cast onto non-woven supports using an adjustable film applicator (Elcometer 3540/4 Four-Sided Film Applicators, Elcometer Ltd., Manchester, UK), with a wet thickness of 100 µm. Finally, the cast membrane underwent coagulation in a pure water bath at a temperature of 28 °C. The membrane was immersed in a pure water bath for a minimum of 1–2 h until it naturally detached from the glass plate after phase separation was fully achieved. The prepared membranes were designated as follows: the pristine membrane was designated as PSF-nAC0 and the composite membranes as PSF-nAC2, PSF-nAC4, PSF-nAC6, and PSF-nAC8. The compositions of the casting solutions used for the fabricated membranes are detailed in Table 1.

### 2.3. Water Contact Angle Test

A 5 μL droplet of pure water was applied to the surface of the membrane by using a micropipette (Zhejiang Lichen Instrument Technology Co., Ltd., Hangzhou, China). The digital microscope captured an image of the water droplet’s contact angle, while CAD software (AutoCAD v2022) computed the angle. We measured the contact angles for each membrane three times and then calculated the average to ensure accuracy.

### 2.4. Filtration Experiments

#### 2.4.1. Water Flux Test

**Figure 1:**
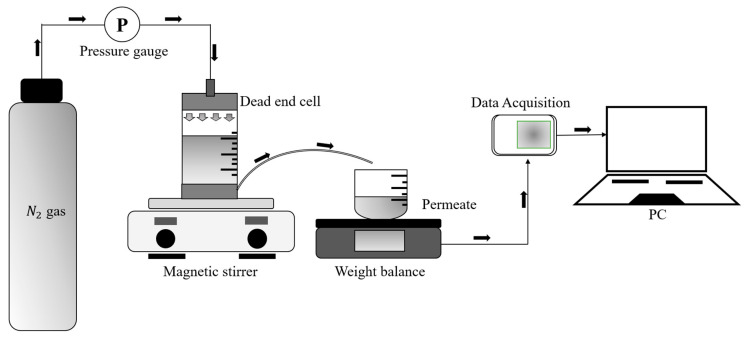
Experimental setup of the water flux experiment.

A water flux experiment was performed utilizing a stirred dead-end cell, as illustrated in Figure 1. The fabricated membranes were trimmed to the necessary dimensions to be applied in the stirred dead-end cell. The feed solution filled the cell, and the entire experiment was conducted at a constant temperature of 28 °C. Nitrogen gas, kept at a specified pressure (2 bar), was delivered into the cell to provide the necessary pressure for the pure water to pass through the membrane’s pores. The weight of the permeate collected in the experiments was used to evaluate the permeated pure water. The quantity of water that permeated the fabricated membrane was meticulously recorded by a data acquisition system during the experiment. The following equations were employed to calculate both the volumetric flux ($J_{v}$) and the permeability.

We used the following equations to calculate both the volumetric flux ($J_{v}$) and the permeability [31].

(1) $$Flux\;(J_{v})=\frac{Q}{A\times ∆t}$$

(2) $$Permeability\;(L_{p})=\frac{J_{v}}{∆P}$$

In the context of membrane analysis, Q represents the volume of permeate water per unit time (L/h), ∆*t* denotes the duration of the sampling period (in hours), *A* signifies the membrane area (measured in square meters), and ∆P represents the operational pressure (in bar).

#### 2.4.2. Protein Separation

A 0.1 wt.% BSA, pepsin, and lysozyme solution was dissolved in a phosphate-buffered saline (PBS) with a pH of 7.2. The subsequent protein rejection test was conducted under an operational pressure of 2 bar. The N4S UV-visible spectrophotometer was used to measure the protein solution’s concentration before and after the test. The rejection of the protein solution (SR) was calculated through rigorous analysis [32].

(3) $$\%SR=\left[1-\frac{C_{p}}{C_{f}}\right]\times 100\;$$

In the context of protein concentration analysis, $C_{p}$ and $C_{f}$ denote the concentrations of the permeated solution and feed solution, respectively.

### 2.5. Membrane Porosity

Porosity of the membrane was investigated to study the impact of introducing activated carbon on membrane pore size, employing the gravimetric method and the following equation [33]:

(4) $$\varepsilon \left(\%\right)=\frac{W_{w}-W_{d}}{\rho H_{2}O\times A\times L}\times 100$$

where A is the active membrane area,  L is the membrane’s thickness, and $\rho H_{2}O$ is the water density (0.998 $g/{cm}^{3}$).

### 2.6. Average Pore Size

The average pore size of the surface membranes was evaluated based on the ultrafiltration test. To calculate the membrane’s average pore size, we utilized the molecular weight of the solute, which exhibited an SR exceeding 80%, using the following equation:

(5) $$\bar{R}=100\left(\frac{\alpha }{\%SR}\right)$$

Here, $\bar{R}$ represents the average pore size, and α denotes the solute radius, which is determined by the Stoke radius obtained from the solute’s molecular weight using Sarbolouki’s method [34].

### 2.7. Molecular Weight Cutoff (MWCO)

The MWCO is characterized by a linear relationship with the pore size of the membrane. When assessing the MWCO of a membrane, the goal is to identify the smallest inert solute that demonstrates protein rejection in the range of 80–100% during an ultrafiltration test [34,35]. In this particular study, BSA, pepsin, and lysozyme were selected as the measured proteins to indicate any potential pore size reductions resulting from the addition of nano-activated carbon powder.

## 3. Results

### 3.1. Membrane Morphology

To elucidate the performance of the fabricated membrane, we examined the surface morphologies using scanning electron microscopy (SEM Phenom ProX, Thermo Fischer, Waltham, MA, USA). SEM is fundamental for assessing membrane morphology. SEM images are routinely employed to investigate membrane surface features, cross-sectional structure, pore distribution, and membrane thickness. These analyses contribute to qualitative assessments of membrane performance.

**Figure 2:**
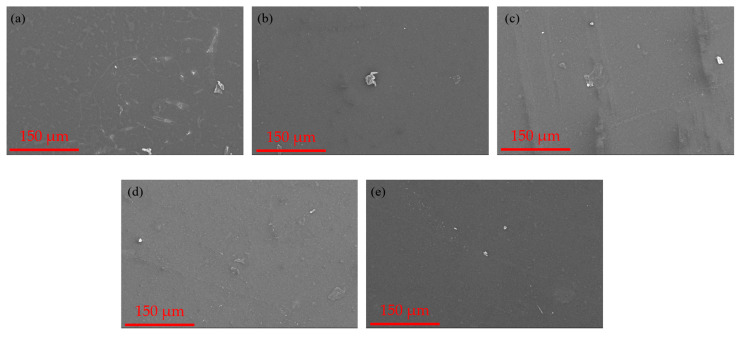
Surface morphology SEM images of the fabricated membranes: (**a**) PSF-nAC0; (**b**) PSF-nAC2; (**c**) PSF-nAC4; (**d**) PSF-nAC6; (**e**) PSF-nAC8.

The SEM images of the fabricated membranes are depicted in Figure 2. The pristine membrane exhibits a smooth top surface devoid of any irregularities. On the other hand, the addition of activated carbon to the membranes altered their morphological structure, resulting in non-uniform distributions and rough surfaces. However, as depicted in Figure 2, the absence of fine cracks on the composite membrane surface suggests that the inclusion of nAC does not compromise membrane strength.

**Figure 3:**
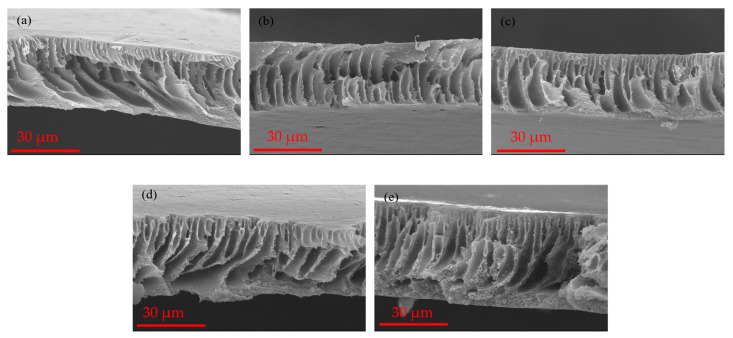
Cross-section SEM images of the fabricated membranes: (**a**) PSF-nAC0; (**b**) PSF-nAC2; (**c**) PSF-nAC4; (**d**) PSF-nAC6; (**e**) PSF-nAC8.

The cross-sectional SEM micrographs of the membranes, as illustrated in Figure 3, reveal an asymmetric morphology characterized by two distinct layers. The top layer is thin and spongy, while the other is a thick, porous sublayer whose shape is similar to a finger-like structure. These layers play distinct roles: the thin layer is responsible for solute permeation, while the thick, finger-like structure layer contributes to solute retention [36].

The formation of these asymmetric membranes occurs during the phase inversion process [37]. Specifically, the polymeric solution in the non-solvent bath precipitates at the interface, leading to the development of a thin skin layer and a porous sublayer characterized by finger-like channels [38]. Surface hydrophilicity plays a crucial role in the distinct behavior of the two porous membranes. It is hypothesized that the exchange rate between solvent and non-solvent during the inversion stage is influenced by both the porosity and the hydrophilicity of the adsorbent [39]. Accelerating the solvent’s diffusion from the cast film into the water bath facilitates the formation of finger-like voids. The substantial adsorption capacity of nAC results in solvent adsorption and enhances the viscosity of the casting solution through effective physical interactions. The inclusion of nAC in the casting solution significantly reduced the solvent diffusion rate, leading to the formation of a mildly porous structure with a morphology resembling that of a sponge, characterized by uniformly smaller voids, as evidenced by the cross-sectional images of the composite membranes [40].

### 3.2. Contact Angle Analysis

**Figure 4:**
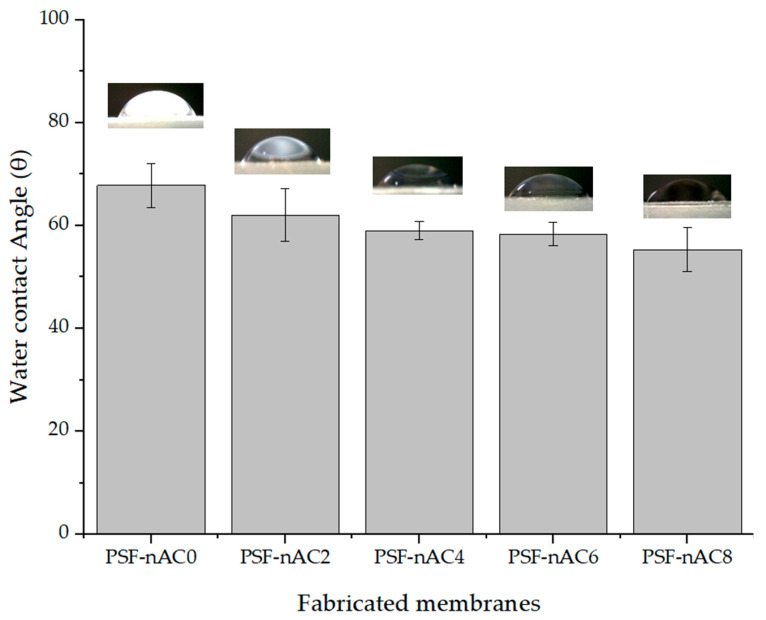
Water contact angle of the fabricated membranes at different levels of nAC concentration.

Hydrophilicity is one of the most crucial factors of a membrane when investigating membrane permeability and understanding the filtration mechanism. The water contact angle of the membranes helps one to analyze the surface hydrophilicity of the fabricated membranes. A hydrophilic membrane generally indicates a lower contact angle, with a value less than 90°. The contact angles of the pristine membrane and composite membranes are presented in Figure 4. As shown in Figure 4, the pristine PSF membrane (0 wt.% nano-activated carbon powder) is found to be the most hydrophobic, whilst the most hydrophilic surface was obtained at a concentration of activated carbon powder of 8 wt.%.

In the membranes containing a nano-activated carbon concentration up to 8 wt.%, the water contact angle values of the membranes decrease with the increase in the concentration of nano-activated carbon. These results state that at a certain concentration, the introduction of activated carbon powder improves the surface hydrophilicity of the membrane. The variation in contact angle with nAC concentration can be attributed to the improved dispersion of nAC within the polymer matrix, allowing water molecules to spread more effectively and resulting in a decrease in the water contact angle [41]. Surface hydrophilicity is crucial for membranes, as hydrophilic surfaces tend to absorb a greater amount of water molecules, resulting in enhanced permeability through the membrane [42].

### 3.3. Pure Water Flux Test Experiments

**Figure 5:**
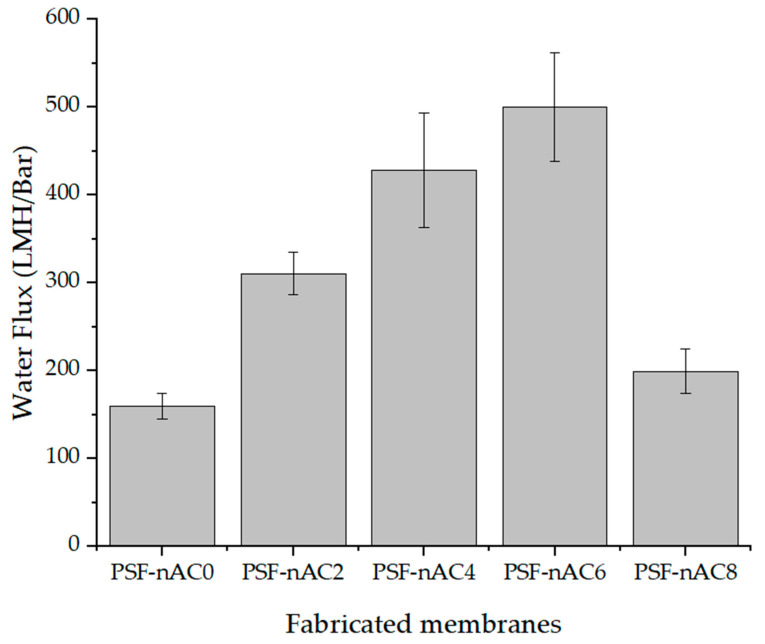
Water flux of the fabricated membranes at different levels of nAC concentration.

Figure 5 illustrates the membrane performance in terms of water flux at varying concentrations of activated carbon powder. Compared to the flux of the pristine PSF membrane (150 LMH/Bar), as the concentration of activated carbon powder increased, the water flux of the composite membrane increased; however, at an 8 wt.% concentration, it began to decrease.

The water flux of the PSF composite membrane achieved the highest value of 500 LMH/Bar with an activated carbon powder concentration of 6 wt.%. The enhanced permeability through the membrane can be attributed to the surface hydrophilicity characteristics of the membranes (as shown in Figure 5), resulting from the incorporation of nAc powder. This finding is in agreement with previous research which stated that the increase in permeability is due to the decrease in the water contact angle [43], since the increased hydrophilicity of the surface is expected to enhance water molecule absorption, resulting in improved membrane permeability. However, when increasing the nAC concentration from 6 wt.% to 8 wt.%, powder aggregation may occur due to the higher amount of powder in the doping solution. Additionally, elevating the nAC concentration in the doping solution led to an increase in the solution’s viscosity, which, in turn, may cause delayed demixing during the wet phase inversion process and result in the formation of powder clots on the membrane’s surface. Optimizing the concentration of activated carbon may lead to improved flux in polymer membranes. Higher concentrations of activated carbon can lead to increased accumulation, weak dispersion, and the formation of disconnected channels, resulting in reduced water flow [44].

### 3.4. Effect of nAC on Protein Rejection

**Figure 6:**
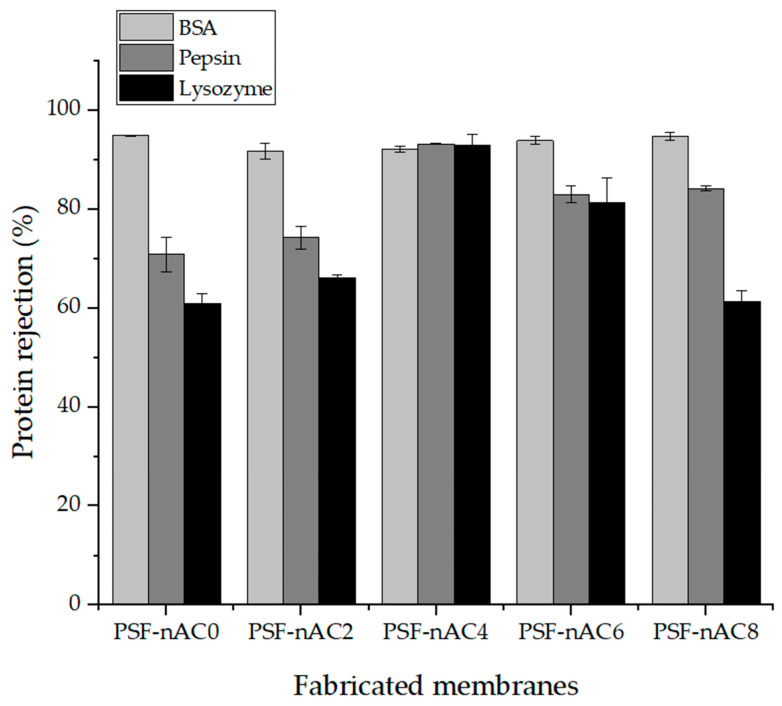
Protein rejection of the fabricated membranes at different levels of nAC concentration.

The protein rejection percentages using the pristine membrane and the composite membrane employing a BSA, pepsin, and lysozyme solution are displayed in Figure 6. In Figure 6, the pristine membrane, not including nAC, exhibits rejection percentages of 94.9% for BSA, 70.8% for pepsin, and 60.9% for lysozyme. The enhanced separation of BSA and the reduced separation of lysozyme can be attributed to their distinct molecular sizes. Moreover, the inclusion of nAC significantly influences the membrane’s rejection performance. As the nAC concentration increased to 6 wt.%, protein separation markedly decreased to 82.9% for pepsin and 81.3% for lysozyme. However, the elevated protein rejection observed in the composite membrane containing 4 wt.% nAC can be ascribed to the reduction in pore size. Protein rejection and permeation ability are primarily influenced by the hydrophobic and hydrophilic interactions of the membrane, rather than its pore size. Consequently, the hydrophilic surface of the membrane impedes the adhesion of hydrophobic protein molecules to the high nAC concentration on the composite membrane surface. Consequently, the composite membrane containing 4 wt.% nAC demonstrated superior rejection performance when compared to both the pristine membrane and the membrane with 6 wt.% nAC. This significant variation in protein rejection can be attributed to morphological changes occurring during the phase inversion method.

Size exclusion, hydrophilic properties, and electrostatic repulsion play pivotal roles in protein molecule separation. In our study, the protein feed solution was prepared at pH 7.2 using PBS, resulting in a negatively charged feed solution. Consequently, electrostatic repulsion significantly contributed to the observed higher protein rejection [45].

Empirical observations reveal that incorporating nanoparticles at higher concentrations leads to a reduction in pore size, enhancing protein rejection. Furthermore, the hydrophilic membrane surface impedes protein attachment, thereby increasing protein rejection [46]. However, elevated concentrations of nAC (6 wt.%) result in accumulation, weak dispersion, and the formation of macrovoids within the composite membrane, ultimately leading to reduced protein rejection [47].

### 3.5. Porosity

The impact of nAC on the modified polymer membrane was assessed by analyzing membrane porosity. The addition of hydrophilic additives led to variations in the performance of the polymer membrane. The porosity values for both pristine and PSF-nAC composite membranes are documented in Table 2.

The porosity of the pristine membrane, in the absence of nAC, exhibited high values (5.78%). The fabricated membrane achieved its lowest porosity (2.78%) when 8 wt% nAC was added. This is due to the fact that upon introducing nAC to the membrane, aggregation within the polymer composition increased, resulting in even higher porosity. Homayoonfal et al. [48] reported a similar finding, demonstrating that the increased addition of superparamagnetic iron oxide nanoadditives to polymeric membranes results in reduced membrane porosity. The decrease in porosity can also be attributed to the elevated viscosity of the membrane solution resulting from solvent and non-solvent demixing during the phase inversion process [49].

### 3.6. Measurement of Average Pore Size

In Table 2, we presented the average pore size for the fabricated membranes. The results indicate that the introduction of activated carbon powder into the membrane matrix led to a reduction in the pore size of the composite membranes. A higher compression of the average pore radius was observed as the activated carbon concentration increased from 4 to 8 wt.% in the case of composite membranes. This behavior aligns with findings reported in the literature [50], where the narrowing of the average pore size can be attributed to the increased heterogeneity inside the structure of the membrane, induced by the introduction of activated carbon powder during the gelatinization period. Interestingly, according to the results shown in Table 2, the average pore size of the membranes increased at 6 wt.% nAC content. In the phase inversion process, as described by Hosseini et al. [51], the presence of nAC reduces the surface tension between pure water (non-solvent) and the polymeric film. This reduction in surface tension enhances the interaction between pure water and NMP (solvent). Consequently, nAC acts as a surfactant, leading to a decrease in polymer concentration on the membrane surface and resulting in the formation of larger pores.

Nevertheless, the average pore size of the composite membranes was smaller than that of the pristine membrane. This discrepancy can be attributed to the fact that the molecular weight cutoff of these composite membranes is either lower or comparable to that of pristine PSF membranes.

### 3.7. Molecular Weight Cutoff Measurement

The molecular weight cutoff measurement for both pristine and PSF-nAC composite membranes, with varying concentrations of nAC, was determined using protein solutions of different molecular weights: 69 kDa for BSA, 35 kDa for pepsin, and 14 kDa for lysozyme. The molecular weight cutoff of PSF-nAC composite membranes, with 2 wt.% nAC, initially measured 69 kDa. However, after adding 4 wt.% nAC, the molecular weight cutoff decreased to 14 kDa, indicating that the modified membranes are suitable for ultrafiltration processes, as displayed in Table 2.

Conversely, the molecular weight cutoff of the membrane increased from 14 kDa to 35 kDa following the addition of 6 wt% nAC. Further increasing the nAC content to 8 wt% resulted in a higher molecular weight cutoff. This enhancement in molecular weight cutoff and mean pore size can be attributed to fluctuations in pore size, which are interconnected with the addition of nanoparticles and the increased viscosity of the doping solution [52]. Table 3 presents a comparison of membrane performance in this study with the results of other works which use PSF as base polymer with a carbon base modifier.

The data in Table 3 showcase the best membrane performance from each cited study. It is evident that PSF membranes with carbon modifiers exhibit varying levels of performance in terms of pure water flux, water contact angle, and porosity. Even when using the same modifier, such as multi-walled carbon nanotubes, the water flux range can be quite broad. This variability may be attributed to the different functionalizing agents and treatments applied to each modified membrane. In this study, the PSF-nAC composite membrane demonstrated high water flux and protein rejection with a hydrophilic surface, making it advantageous for protein separation and water treatment applications.

## 4. Conclusions

The impact of incorporating activated carbon on improving the properties and performance of composite PSF membranes was thoroughly investigated, alongside the determination of the optimal concentration for promoting filtration capabilities of the membrane. The addition of activated carbon powder led to favorable changes in several key properties of the PSF membrane, including the average pore size, porosity, water flux, protein rejection, and surface morphology. Notably, the water flux experienced a nearly 200% increase at an activated carbon concentration of 4 wt.%. In addition, the surface of the membrane became more hydrophilic at this concentration, with the water contact angle being reduced by 15°. In the lysozyme separation tests, a concentration of 4 wt.% activated carbon in the membrane exhibited the highest rejection rates (93–95%). In summary, the experimental findings highlight the potential of activated carbon powder as a prominent additive for extending the application of PSF membranes in protein separation processes.

## Figures and Tables

**Table 1:** Composition of the fabricated membranes.

Membrane Code	Polysulfone (wt.%)	nAC (wt.%)	NMP (wt.%)
PSF-nAC0	20	0	80
PSF-nAC2	20	2	78
PSF-nAC4	20	4	76
PSF-nAC6	20	6	74
PSF-nAC8	20	8	72

**Table 2:** Porosity pore radius and MWCO of the fabricated membranes.

Membrane Code	Porosity (%)	Pore Radius, $\bar{R}\;(A{}^{\circ})$	MWCO (kDa)
PSF-nAC0	57.8	40.43	69
PSF-nAC2	52.2	41.76	69
PSF-nAC4	34.3	19.57	14
PSF-nAC6	34.5	22.22	35
PSF-nAC8	27.8	29.76	35

**Table 3:** Comparison of PSF membranes with various carbon modifiers.

Modifier	PWF (LMH/bar)	BSA Rejection (%)	WCA	Porosity, (%)	Pore Radius, $\bar{R}\;(nm)$	Ref
PVP + multi-walled carbon nanotubes (MWCNT)	12.8	-	59	54.57	3.015	[53]
Carboxyl MWCNT (mMWCNT)	48	-	73	-	2.55	[54]
PVP + carbon–charcoal nanomaterial	196.3	95.8	69.6	91	13	[55]
Carboxilated MWCNT (β-CD-cMWCNTs)	320	67.5	-	87	10.1	[56]
MWCNT coated with polydopamine	174	99.21	42.85	35.7	-	[57]
PVP + carbon dots + silica	7.4	-	75.6	89.88	6.5	[58]
Nano-activated carbon	428	92.1	59	34.3	1.95	This work

## Data Availability

The original contributions presented in the study are included in the article, further inquiries can be directed to the corresponding author.
